# Quantitative Microbial Risk Assessment Based on Whole Genome Sequencing Data: Case of *Listeria monocytogenes*

**DOI:** 10.3390/microorganisms8111772

**Published:** 2020-11-11

**Authors:** Patrick Murigu Kamau Njage, Pimlapas Leekitcharoenphon, Lisbeth Truelstrup Hansen, Rene S. Hendriksen, Christel Faes, Marc Aerts, Tine Hald

**Affiliations:** 1 Research Group for Genomic Epidemiology, Division for Global Surveillance, National Food Institute, Technical University of Denmark, 2800 Lyngby, Denmark; pile@food.dtu.dk (P.L.); rshe@food.dtu.dk (R.S.H.); tiha@food.dtu.dk (T.H.); 2 Research Group for Microbiology and Hygiene, National Food Institute, Technical University of Denmark, 2800 Lyngby, Denmark; litr@food.dtu.dk; 3 Interuniversity Institute for Biostatistics and Statistical Bioinformatics, Hasselt University Katholieke Universiteit Leuven, 3590 Diepenbeek, Belgium; christel.faes@uhasselt.be (C.F.); marc.aerts@uhasselt.be (M.A.)

**Keywords:** quantitative microbial risk assessment, whole genome sequencing, exposure assessment, predictive modeling, machine learning, finite mixture models, *Listeria monocytogenes*

## Abstract

The application of high-throughput DNA sequencing technologies (WGS) data remain an increasingly discussed but vastly unexplored resource in the public health domain of quantitative microbial risk assessment (QMRA). This is due to challenges including high dimensionality of WGS data and heterogeneity of microbial growth phenotype data. This study provides an innovative approach for modeling the impact of population heterogeneity in microbial phenotypic stress response and integrates this into predictive models inputting a high-dimensional WGS data for increased precision exposure assessment using an example of *Listeria monocytogenes*. Finite mixture models were used to distinguish the number of sub-populations for each of the stress phenotypes, acid, cold, salt and desiccation. Machine learning predictive models were selected from six algorithms by inputting WGS data to predict the sub-population membership of new strains with unknown stress response data. An example QMRA was conducted for cultured milk products using the strains of unknown stress phenotype to illustrate the significance of the findings of this study. Increased resistance to stress conditions leads to increased growth, the likelihood of higher exposure and probability of illness. Neglecting within-species genetic and phenotypic heterogeneity in microbial stress response may over or underestimate microbial exposure and eventual risk during QMRA.

## 1. Introduction

Microbial risk assessment (MRA) has been adopted as a framework to enable weighing of options for public health protection and mitigation of the impact of exposures to microbial hazards [1,2]. MRA involves the systematic determination of the risk associated with microbial hazards in a food with an objective of characterizing the nature and likelihood of harm resulting from human exposure to these microbial agents through food consumption [2]. The level in terms of the prevalence and concentration of a pathogen ingested through consumption of a serving of a food are determined in one of the three MRA steps referred to as exposure assessment.

**Figure 1:**
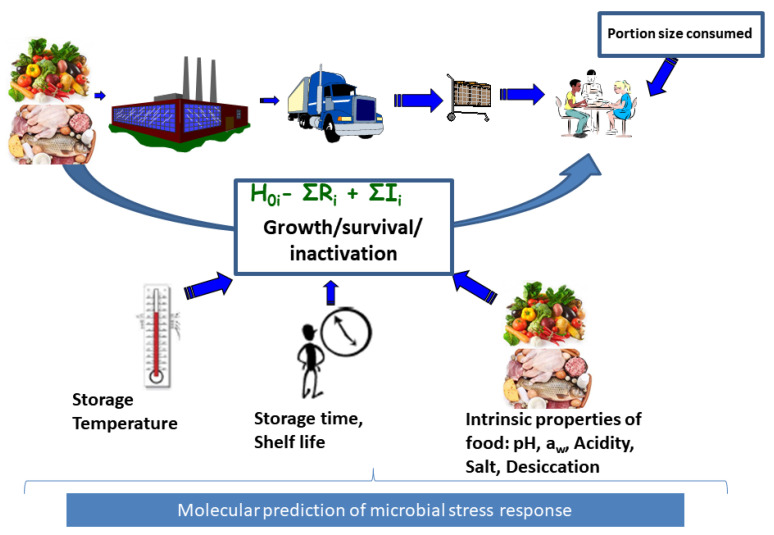
Food chain exposure assessment. This involves input data consisting of food consumption data and microbial growth data together with associated food inherent, environmental and process induced factors influencing microbial growth (I) or reduction (R) in any of the stages of the food chain including processing, distribution, retail and consumer level. The final concentration at exposure consists of initial contamination (Ho) plus the total increase (∑I), minus total reduction (∑R). Use of molecular data will support exposure assessment for strain or microbial population subgroup *i*.

Exposure assessment involves assessing the growth, survival and inactivation of the microorganisms from farm to fork in order to compute the final microbial concentration a consumer is exposed to in a food serving while incorporating data on quantities consumed (Figure 1). If the MRA is conducted based on available consumer level food samples, direct assay of microbial concentration is possible at the point of consumption. However, this is often not the case and it becomes expedient to model and project the impact of changes in conditions that may influence growth and inactivation of the microorganisms starting from the concentration determined from foods samples from other farm to fork steps. Such modeling is supported by availability of microbial concentration data at the point of contamination such as farm, distribution, processing and retail levels of the value chain. It is therefore desirable to model changes in microbial concentrations between the point of contamination and human exposure to the pathogen, a concept termed as predictive microbiology [3] (Figure 1). In predictive microbiology, “primary models” describe changes in microbial concentration with time. The aim of primary models is to estimate kinetic parameters describing either inactivation rate or the typical four phases of microbial growth which include the lag phase, maximum growth rate and maximum population density [4]. Lag phase is an adaptation period where bacterial cells adjust to a new environment after which they grow exponentially at the maximum growth rate ($\mu_{max}$) until growth reaches a plateau at the maximum population density also referred to as the stationary phase [4]. These post-contamination growth or inactivation changes in microbial concentration are influenced by food processing and storage environment conditions such as pH, organic acids, water activity (influenced by desiccation and salt concentration) and temperature. The impact of these conditions on microbial growth and/or inactivation can be described by secondary models (Figure 1) [4].

The introduction of high-throughput DNA sequencing technologies (WGS) has made possible the high resolution typing and study of bacteria at the strain level. The rapid drop in costs of WGS has seen this approach incorporated as a standard surveillance technique in the high resolution subtyping of strains for epidemiological purposes and a powerful tool in decision making during outbreak investigations [5]. However despite the potential of this technology, the application of WGS data in microbiological risk assessment has only been a subject of increasing discussion but remains a vastly unexplored area in the public health domain [5].

The European Food Safety Authority (EFSA) recently made a comprehensive scientific opinion concerning the use of whole genome sequencing for risk assessment of food-borne microorganisms [6] while den Besten et al. reviewed the potential use of omics data for exposure assessment [7]. WGS data has shown potential in predicting the potential for microbial growth or survival in the food value chain and eventually in the host [6]. WGS data could assist in unraveling biological variability which induces a diverse response in microorganisms to differing environmental conditions [8]. Strains within a given bacterial species differ in their phenotypic characteristics such as variation in abilities to grow or survive under conditions encountered in foods from farm to fork [6]. Variability in microbial growth and/or inactivation may emanate from physicochemical properties of the food, processing conditions or parameters and natural variation between individuals within the same microbial population [7] (Figure 1). Genetic changes may result in large phenotypic differences in growth, survival and inactivation of microorganisms [9]. Ignoring such changes in a seemingly homogeneous microbial population which assumes population average behavior may result in over- or underestimation of microbial exposure and associated risk [10]. WGS therefore provides the potential to incorporate microbial strain variability in the identification of “high risk” bacterial subpopulations and their distribution among the whole population [7] assuming that specific genetic determinants commonly occur in all such sub-populations. This will assist in fine tuning of exposure assessments. Carlin et al. [11] reported variability in cardinal growth parameters of six genetic groupings of *Bacillus cereus*. Berendsen et al. [12] reported two distinct groupings in heat resistance of bacterial spores which could be attributed to mobile genetic elements. Such genetic elements could therefore function as predictors/biomarkers discriminating between different levels of resistance to stress conditions such as heat.

Application of genotypic data for exposure assessment has been hindered by challenges in the translation of high dimensional WGS data into reduced phenotypic information with a resultant metric that is useful in MRA [13]. Loss of biological meaning or important genetic predictors may result when data reduction methods are applied [14]. Approaches such as network analysis [15] and machine-learning algorithms [14,16,17] are a family of techniques that solve the problem of predictive modeling in cases of highly dimensional, heterogeneous datasets with complex relationships between the predictors and the outcomes and to derive fewer features (e.g., genes) important for these predictions. Incorporating WGS data in predictive modeling to draw conclusions beyond data obtained will foster models supporting reduced need for frequent use of slow culture dependent laboratory tests and food validation of growth, survival and inactivation models under differing conditions.

The other challenge involves attempts to resolve grouping of species into subgroups based on their phenotypic stress response data such as growth or inactivation rate, the lag time and the maximum population density. Some strains may be extra tolerant, some moderately tolerant, while some may exhibit varying levels of susceptibility to process and storage conditions. This presents evidence of unobserved heterogeneity in stress response. Most studies group strains into species average responses and risk assessment efforts rely on reported historical studies to define growth or inactivation parameters which may lead to modest stress tolerance or susceptibility reported in many predictive microbiology studies. This can be misleading because such average effects may be a mixture of substantial subgroups each with its subgroup specific proportion of strains and average growth parameters for each subpopulation in the data. A befitting approach for this kind of analysis is finite mixture models [18]. This will assist in predicting pathogen behavior variability due to heterogeneity in physiological state and stress response [7].

The aim of this study was to model the impact of population heterogeneity in microbial phenotypic stress response and integrate this into predictive models inputting WGS data for increased precision quantitative exposure assessment using an example of *L. monocytogenes*. The application of the approaches from this study was demonstrated using an example of consumer level quantitative microbial risk assessment (QMRA) to predict influence of different stress response subgroups of *L. monocytogenes* on risk of illness from consumption of cultured milk in three consumer groups. Scenario analysis after QMRA was used to illustrate the possibility that a QMRA assuming that bacteria grows as a single population characterized by an average growth rate value may either over- or underestimate the risk.

## 2. Materials and Methods

### 2.1. Methodology Outline

The first part of this study derived evidence that within each taxonomic unit population, there are sub-populations differing in proportions and ability to grow under different stress conditions. Finite mixture models were used to answer questions concerning: how many of *g* sub-populations or components can be distinguished for each stress type? What underlying stress response category of each of the *g* sub-populations represent its mean and variance? What are the relative proportions of strains in each sub-population? The second aim of this study was to select machine learning predictive algorithms inputting highly dimensional WGS data to predict into which of the *g* sub-populations that new strains with unknown stress response data can be categorized. If the sub-population is predicted for new strains, the proportion of each sub-population can then be calculated while the mean and variance of the $\mu_{max}$ or LPD for each of these populations is already computed from the finite mixture models. The final aim was to illustrate the application and importance of the approaches derived in the two previous objectives. QMRA was conducted with cultured milk products at consumer level as an example to: predict stress phenotype components for new unknown strains given their WGS data; estimate the probability of illness for three consumer sub-populations and the number of expected cases per million consumers; and conduct scenario or sensitivity analysis to assess influence of changes in proportion of strains in each stress phenotype component on risk of illness.

**Figure 2:**
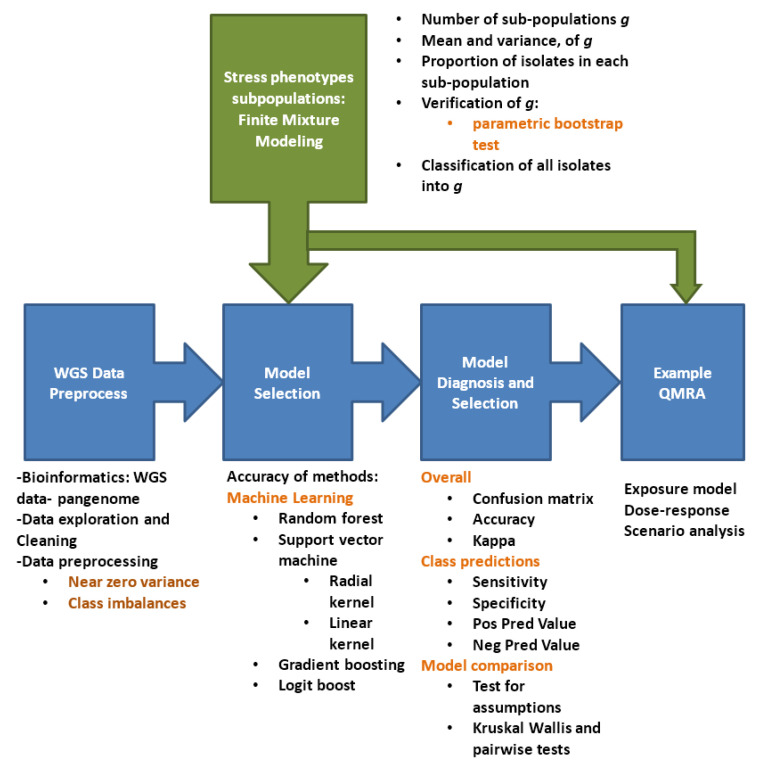
Methodology flow diagram.

Figure 2 summarizes the methodology steps followed in this study.

### 2.2. Hazard Identification

*L. monocytogenes* is a ubiquitous Gram-positive bacterium that causes listeriosis. Listeriosis is characterized by severe symptoms including septicemia and meningitis especially in highly susceptible groups such as newborn children, pregnant women, the elderly and immunocompromised patients [19]. Listeriosis occurs at low frequencies but with high fatalities thereby ranking *L. monocytogenes* as a food-borne pathogen of high concern. A vast majority of the cases (as high as 99%) have been attributed to contaminated food [20].

Genetic variants or subtypes of *L. monocytogenes* referred to as strains have exhibited substantial variation in virulence and environmental stress resistance [21]. Attempts to address this between strain variation in virulence and stress resistance have focused on serogroups in most studies. There has been some success in demonstrating the role of particular serogroups in increased number of sporadic cases and outbreaks [22,23]. However, it has been recognized that serotypes from foods poorly reflect disease distribution [21]. WGS rather than serotype data have therefore been proposed for higher resolution studies on virulence and environmental stress resistance targeting genetic strain-specific level of evidence [22]. Use of WGS data will be an important step towards increased understanding and improved control efforts to address increase in disease incidence due to the emergence of single or combinations of new virulence and environmental stress resistance genetic elements associated with *L. monocytogenes* [21]. Use of WGS data also presents a chance to improve public protection and mitigation of impact of such exposures to microbial hazards through higher resolution MRA efforts.

### 2.3. *L. monocytogenes* Strain Data

A collection of 166 *L. monocytogenes* strains from Canada and Switzerland, as well as associated data including WGS data and growth phenotypes during different stress conditions, were obtained from a previous study by Hingston et al. [24]. This panel of strains consisted of strains obtained from food and food processing environment strains from Canada (n = 139) and Switzerland (n = 20), six strains associated with sporadic human listeriosis cases and an asymptomatic human case from Switzerland.

Hingston et al. [24] sequenced the strains and further evaluated their growth characteristics under cold (4 ${}^{{}^{\circ}}$C), salt (6% NaCl, 25 ${}^{\circ }$C), desiccation (33% RH, 20 ${}^{\circ }$C) and acid (pH 5, 25 ${}^{\circ }$C) stress conditions. Growth parameters including relative lag phase duration (LPD), relative maximum growth rate ($\mu_{max}$) and maximum cell density ($N_{max}$) were studied for all the strains.

### 2.4. Bioinformatics

*L. monocytogenes* genomes were assembled and processed into a matrix of percent similarity between all genes (pangenome) converted to amino acid sequences from the *L. monocytogenes* genomes. In summary, gene families were obtained through determination of predicted genes in amino acid sequences based on the assembled genomes of the *L. monocytogenes* dataset using Prokka software. Prokka was used to annotate and predict genes. Predicted genes based on the assembled genomes of the *L. monocytogenes* dataset were aligned all-against-all using Roary, the pangenome pipeline used to identify gene clusters and the pangenome [25]. The intersection of gene clusters common to all the genomes from all the strains was used to define the core genes and the accessory genes were defined as a complementary of the core genes i.e., those gene families that were not part of the core genes. The pangenome sequences in the form of amino acid sequences were retrieved from Roary output. A matrix of percent similarity between the genes in the pangenome and the *L. monocytogenes* genomes was generated using TBLASTN, a Basic Local Alignment search tool (NCBI-blast version 2.2.31+) [26]. This matrix was used as input for predictive models.

### 2.5. Finite Mixture Modeling

#### 2.5.1. Designation of Stress Response Phenotype Components

**Figure 3:**
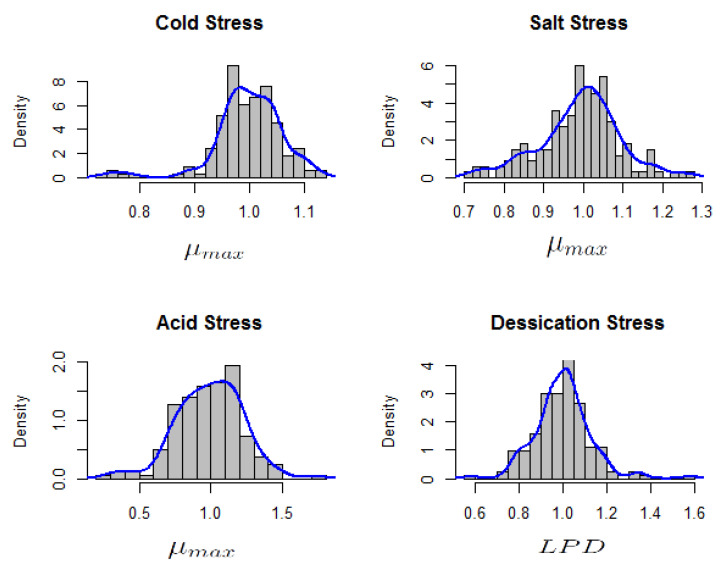
Histogram of relative maximum growth rates ($\mu_{max}$) for acid, cold and salt stress response and relative lag phase duration (LPD) of desiccation stress response in 166 *L. monocytogenes* strains.

Interest was in the relative $\mu_{max}$ for cold, salt and acid stress while the focus was on relative LPD for the case of desiccation stress survival. Exploration using histograms indicated multi-modality (Figure 3). Such multi-modality suggests the presence of some underlying or latent groups whose structure is unknown.

Stress response categories cannot be reliably described by assuming stress category cut-offs which assume single distributions. There seemed to be underlying categories which correspond to stress tolerance classes. Within each of the tolerance class, a rather ‘homogeneous’ distribution seems plausible such that the relative areas of the local densities at the modes give an indication of the proportion of the strains in that particular tolerance class. Such mixed populations consisting of a joint distribution over observed and latent variables may be interpreted into their simpler components using finite mixture models [18]. Assuming the population *P* of the strains is composed of *g* sub-populations, $P_{1},P_{2},\ldots ,P_{g}$, the questions of interest would be: how many of the *g* sub-populations can be distinguished for each stress type, what underlying stress response category they represent and what are the relative proportions of strains in each sub-population? Each of the sub-populations $P_{j}$, represents a proportion $\pi_{j}$ of the total population with the constraints: $\sum_{j=1}^{g}\pi_{j}=1$ and $0\leq \pi_{j}\leq 1$. Letting *X* indicate the population from which an observation has been sampled, the distribution of *X* is discrete consisting of support {1,2,…,g} with corresponding probabilities $\left\{\pi_{1},\pi_{2},\ldots ,\pi_{g}\right\}$: X∼$\left(\begin{array}{c} 1\;\;2\ldots \;\;g\\ \;\;\pi_{1}\;\pi_{2}\ldots \pi_{g} \end{array}\right)$. *X* is considered latent because it has not been observed [18]. The density of outcome *Y* (relative LPD or $\mu_{max}$ in this study) in sub-population $P_{j}$ from the entire population of strains *P* is equal to: $f\left(y\right)=\sum_{j}f\left(y|X=j\right)P\left(X=j\right)=\sum_{j}\pi_{j}f_{i}\left(y\right)$. The distribution of *Y* is termed as a finite mixture with *g* components [18]. In our case, the density of relative (LPD) or relative ($\mu_{max}$) for L.monocytogenes is a stochastic variable $Y|\mu ∼N(\mu ,\sigma^{2})$ such that: μ∼$\left(\begin{array}{c} \mu_{1}\;\;\mu_{2}\ldots \mu_{g}\\ \pi_{1}\;\;\pi_{2}\ldots \pi_{g} \end{array}\right)$ with unknown number of *g* components. The interest in this study was to find out: how many components or sub-populations $P_{j}$, what are the components and their relative proportions $\pi_{j}$?

In order to determine the number of mixtures or the *g* sub-populations, one may either assume knowledge of the number of sub-populations and thereby consider the number *g* of components to be fixed or known. However, a more realistic approach to avoid subjective selection of *g* is to treat it as a parameter in the likelihood, and to estimate it from the available data. This involves the use of Non-Parametric Maximum Likelihood Estimation (NPMLE) which, unlike classical ML Estimation theory, does not consider the number of parameters in the likelihood as fixed. NPMLE involves the use of Expectation–Maximization algorithm initially designed for Maximum Likelihood Estimation in situations with missing data. In our situation, we consider the underlying latent variable *X* involving the component membership as missing. The number of components *g* is treated as a parameter in the likelihood which is estimated from the available data.

The Expectation–Maximization (EM) algorithm commonly converges to local maxima depending on the starting values. In order to initially estimate the potential number of sub-populations in the data, the vertex exchange method (VEM) algorithm was applied [18]. VEM algorithm is flexible for the support size and provides starting values for the EM algorithm. The EM algorithm which involves fixed support size will in this way be initiated with starting values very close to the global maximum which ensures proper convergence of the EM algorithm to a global maximum [18]. This avoids convergence of the EM algorithm to a local maximum, an aspect which is dependent on starting values. Both Phases 1 and 2 were computed in the *R* package CAMAN.

The initial solution of the combination of the VEM and the EM algorithms may sometimes overestimate the number of components *g*. The appropriateness of the number of components computed using the VEM and EM algorithms was checked using parametric bootstrap simulating from a mixture model under the null hypothesis with $g=g_{o}$ components [18]. To diagnose whether the solution for $\hat{G}$ is the NPMLE, the gradient functions d(G,p) for the mixing distribution *G* were plotted and conditions were checked (Supplementary File, Section S1).

#### 2.5.2. Classification of *L. monocytogenes* Strains into Components of the Mixture

After fitting of the mixture models, the next step was classification of the strains into the different mixture components to indicate what component of the mixture each strain is most likely to belong to. This is done based on posterior probabilities. Defining indicators $Z_{ij},i=1,\ldots ,N,j=1,\ldots ,g$ as:

$$Z_{ij}=\left\{\begin{array}{cc} 1, & \mathrm{if}\;\mathrm{observation}\;i\;\mathrm{belongs}\;\mathrm{to}\;\mathrm{component}\;j\\ 0, & \mathrm{otherwise}. \end{array}\right.$$

We can define the component probabilities $P\left(Z_{ij}=1\right)=\pi_{j}$, which are referred to as the prior probabilities [18]. These probabilities express how likely it is for the *i*-th strain to belong to component *j*, without taking into account the observed response value $y_{i}$ for that strain. The posterior probability for strain *i* to belong to the *j*-th component is then:

$$\pi_{ij}=P\left(Z_{ij}=1|y_{i}\right)=\frac{f_{i}\left(y_{i}|Z_{ij}=1\right)P\left(Z_{ij}=1\right)}{f_{i}\left(y_{i}\right)}=\frac{\pi_{j}f_{ij}\left(y_{i}\right)}{\sum_{j}\pi_{j}f_{ij}\left(y_{i}\right)}.$$

This posterior probability $\pi_{ij}$ therefore expresses how likely it is for the *i*-th strain is to belong to component *g*, taking into account the observed response value $y_{i}$ for that strain. $\pi_{ij}$ depends on the unknown parameters $\pi_{1},\ldots ,\pi_{g}$ and the general θ vector which can be replaced by their estimates from the fitted mixture model. The classification rule followed involved classifying strain *i* into component *j* if and only if $\pi_{ij}=max_{k}\left\{\pi_{ik}\right\}$ which indicates classifying into the component to which the strain *i* is most likely to belong [18].

### 2.6. Predictive Modeling

Figure 2 summarizes the methodology steps followed during the predictive modeling. The aim was to predict growth at sub-population level using the genetic composition of the *L. monocytogenes* strains. This is under the hypothesis that the machine learning models can recognize certain genetic patterns from the input data and use this to predict the stress phenotype (relative $\mu_{max}$ and LPD) in a sample whose stress phenotype is unknown but whose genetic sequencing data are presented. Figure 2 illustrates the steps followed in the machine learning predictive modeling.

Supervised learning was conducted to classify patterns in the WGS predictor dataset (also referred to as instances or features) into a set of categories (also referred to as classes or labels) represented by the stress phenotype components. The aim was for each of the stress types to perform classification of new strains into one of the stress response categories defined using finite mixture modeling by using a ML model trained from a WGS training set of the data such that: $y_{c}=f_{c}\left(X,\theta_{c}\right),y_{c}\in Ƶ$ [27], where *X* is the WGS data vector for the new strain, $y_{c}$ is the category where the new observation belongs, $f_{c}\left(.\right)$ is the classification function we are interested in training, $\theta_{c}$ is the parameter set for $f_{c}\left(.\right)$ and Ƶ is the set of stress response categories. Interest may be for instance to classify an *L. monocytogenes* strain which is highly salt stress tolerant ($y_{ht}$) from the possible set of stress tolerance categories (Ƶ) susceptible, tolerant and highly tolerant. This classification function $f_{c}\left(.\right)$ can be used to predict the stress response category for an *L. monocytogenes* strain whose stress phenotype is unknown given the WGS data for the new strain.

#### 2.6.1. Data Exploration

Some of the predictors may contain single unique values and are referred to as zero variance predictors [28]. Such predictors may not be useful for splits in tree-based models because they add no or little extra information. This situation also applies for those predictors that have only a few unique values at very low frequencies which could be termed as near zero variance predictors [28]. WGS predictors were explored for zero and near zero-variance predictors as proposed by Kuhn and Johnson [28]. These were removed from the dataset as they have unique values at low frequencies and may during subsequent splitting of the data into cross-validation/bootstrap subsamples result in propagation of zero and near zero-variance predictors subsequently resulting in model fit instabilities [28].

Considerable imbalances in number of samples in each category of stress response (e.g., tolerant or susceptible to acid) (Table 2). In such imbalanced data, poor class specific performance may result due to bias in model training process towards important patterns in the predictors associated with the larger classes [29]. Categories with the lowest number of samples had a considerably low number of samples and up-sampling was performed where strains from the minority classes were sampled with replacement until each category had approximately the same number [30].

#### 2.6.2. Models

Ensemble methods consist of powerful prediction model choice in cases of complexities arising from dimensionality and structure of the dataset or relationship between the predictors and outcomes. Multiple weighted models are aggregated which results in a unit model outperforming the constituent single models [27]. Examples of ensemble approaches include bootstrap aggregation (bagging), adaptive boosting (boosting) and random forest, decomposition methods, negative correlation learning methods, multi-objective optimization-based ensemble methods, fuzzy ensemble methods, multiple kernel learning ensemble methods and deep learning-based ensemble methods [27]. Different algorithms possess potentially useful characteristics depending on the type of data. ML models were evaluated from algorithms commonly used in genetics including random forest (RF), support vector machine (SVM) (radial and linear kernels), neural network (NN), stochastic gradient boosting (GB) and logit boost (LB) [31,32,33,34]. These models are more likely to produce empirically optimum results yielding most accurate models across many problem domains [28]. RF is characterized by good performance in situations like this study where the number of predictors far exceed that of samples. RFs are robust in the case of predictors characterized by weak effect, high correlations and the presence of interactions. RF provide adequate accuracy for simple and complex classification situations and have modest fine-tuning requirements for parameters and no distributional assumptions for the predictor variables [28]. RF further improves on the advantages of bagged trees by decorrelation of the trees [35]. For each of the decision trees built in a similar fashion to bagging, a random sample of *m* predictors is chosen as split candidates from the full set of predictors and the split is allowed to use only one of those *m* predictors. A new set of *m* predictors is selected at each split where the number of *m* is approximately equal to the square root of the total number of predictors. This prevents the problem of correlation where bagged trees look quite similar to each other [35]. SVM models present training data as points in space, which are mapped so that the data from separate categories are divided by a clear gap by making this gap as wide as possible [36]. SVMs apply mathematical features that highly adapt them for the highly dimensional genetic data such as the flexibility in choosing a similarity function, sparseness of solution for large data sets, aptness for large feature spaces and the capacity to recognize outliers [37]. In support vector machines, the prediction equation is only a function of the training set samples that are closest to the boundary also termed as the support vectors [35]. These support vectors contain only the information necessary for classification of the new samples [35]. SVM is a modeling choice which is powerful, highly flexible and robust to outliers [31]. The SVM method has the advantage of applying kernel functions of inner products of predictors by arraying predictors in the observation space using a set of inner products [38]. This helps in dealing with data complexities where classes cannot be easily separable by a hyperplane [35]. LB and GB emanate from the boosting family of algorithms [39,40,41]. Boosting functions by coalescing or boosting a number of weak classifiers (defined as classifiers that predict only marginally better than random) into an ensemble classifier characterized by superior generalized classification accuracy [28]. Earlier boosting algorithms included AdaBoost, which led to later versions including Friedman’s stochastic gradient boosting. GB has many properties in common with RF such as robustness to outliers, missing data and presence of correlated and less important variables. Neural networks are powerful nonlinear regression techniques emanating from concepts mimicking mode of operation of the brain [42,43]. The outcomes are modeled by an intermediary set of variables which are not observable as in the case of partial least squares. These unobserved variables are referred to as hidden variables or units and they are linear combinations of the original predictors which, in contrast to partial least squares models, are not estimated in a hierarchical manner [28]. Each of the hidden units consists of linear combination of some or all the predictors, which is transformed by a nonlinear function *g*(·), for instance a sigmoidal function [28]. All analyses were conducted in *R* Version 3.5.1, according to the codes in the Supplementary file (Section S4).

#### 2.6.3. Model Selection

The predictive performance was evaluated on models generated from k=10-fold cross- validations [28,44].

#### 2.6.4. Model Evaluation

Accuracy scores were calculated from the confusion matrix based on balanced accuracy which calculates a posterior distribution rather than averaging the accuracy over the 10-fold cross-validations [45]. To further interpret the accuracy of class distributions from the confusion matrix for each of the models, Cohen’s Kappa (κ) was used. Zero κ values can be interpreted as no agreement between the observed and predicted classes, while values of one suggest perfect agreement. To interpret κ values, Landis and Koch [46] suggested values of “0–0.20 = slight”, “0.21–0.40 = fair”, “0.41–0.60 = moderate”, “0.61–0.80 = substantial” and “0.81–1 = almost perfect”. An alternative interpretation was proposed by Fleiss et al. [47] who suggested that κ values greater than 0.75 are excellent, 0.40–0.75 are fair to good and <0.40 are poor. Sensitivity, specificity, positive predictive value and negative predictive values associated with the prediction of stress response categories were also computed.

Statistical hypothesis tests were used to evaluate if differences in mean accuracy from the 10-fold cross-validations between the algorithms for each stress type were significant. Due to violations in analysis of variance (ANOVA) assumptions, [48] the non-parametric Kruskal–Wallis test was conducted.

Bias-corrected and accelerated bootstrap (BCa) confidence intervals (CI) for the mean accuracies of each model were calculated using 1000 simulations [49]. These CIs are second-order, in contrast to the percentile intervals which are “first-order” intervals computed from quantiles of the bootstrap distribution [49].

### 2.7. Example Application of Concept

The impact of *L. monocytogenes* stress response heterogeneity on the risk of illness in three consumer groups attributable to consumption of cultured milk was modeled as an example illustrating the potential of the approaches proposed in this study.

#### 2.7.1. Prediction of *L. monocytogenes* Stress Response Components

The first step involved the classification of new strains of food origin whose stress phenotype is unknown into one of the stress response components defined using finite mixture modeling (using ML models selected from Section 2.6). Acid, cold, desiccation and salt stress response phenotypes were predicted for a set of 201 *L. monocytogenes* strains previously whole genome sequenced. These strains originated from dairy products (n = 37), fish (n = 35), mixed food types (n = 28), meat (n = 44), ready-to-eat foods (n = 31) and vegetables (n = 26). Final models selected from Section 2.5 above were used for prediction of the unknown stress response categories of these new strains whose WGS matrix of predictors was derived as described in the bioinformatics Section 2.4.

#### 2.7.2. Quantitative Microbial Risk Assessment

The inherent or processing induced physico-chemical characteristics of a food such as the stress factors studied here influence microbial growth, survival or inactivation. For instance, fermentation lowers the pH which increases acidity of cultured milk products to around 4.6 in cultured dairy products due to lactic acid fermentation in products such as yogurt, buttermilk and sour cream [22]. The strains in the acid stress response study by Hingston et al. [24] were tested for tolerance of pH 5 which is slightly higher than that of cultured milk products (pH 4-4.6), which is therefore within the range invoking the need for stress adaptation in *L. monocytogenes*. These are popular dairy products highly consumed by a large proportion of the population. Supplementary Table S1 from our predictions indicated about half of strains associated with dairy products would be acid tolerant. Quantitative microbial risk assessment on cultured milk products is therefore an interesting case to illustrate the phenotype class prediction and role of *L. monocytogenes* strain heterogeneity in differing risks of illness depending on the proportion of different sub-populations of stress response components.

Table 1 shows a summary of the quantitative risk assessment model for illness after exposure to cultured milk containing *L. monocytogenes* including variables, equations or distributions of the input parameters and data sources. There is scarcity of direct data on *L. monocytogenes* consisting of both WGS and microbial concentrations in various food products. In this risk assessment, quantitative data such as microbial concentrations were obtained from a large scale *L. monocytogenes* risk assessment by FDA et al. [22].

Data for initial contamination, consumer storage time and portions consumed per serving was obtained from FDA et al. [22] and the distribution was estimated by random sampling from uniform, pert and log normal distributions, respectively depending on the available data. The mean and variance of the relative $\mu_{max}$ from the two components of the acid stress response were used together with consumer storage period as inputs for the exponential phase of the three-phase linear model [50] to determine increase during holding. SVMR predicted that 50% of the *L. monocytogenes* strains would be acid stress susceptible growing at a reduced relative $\mu_{max}$ of 0.762±0.047 while the other half would be resistance growing at a higher relative $\mu_{max}$ of 1.007±0.047. These relative $\mu_{max}$ values and proportion of strains belonging to each of the two mixture component were taken into account when calculating increase during holding. It is assumed that the contamination in each serving will contain both tolerant and susceptible *L. monocytogenes* at this proportion of 50%. The final dose consumed per serving was calculated from the quantity consumed per serving and the total number of organisms consumed. The number of organisms consumed were a sum of the initial number and the increase due to growth during storage. This served as input for the exponential dose–response model [51]. The dose–response parameter *r* of the exponential dose–response model indicates the probability of a single bacterial cell to cause listeriosis. This *r* parameter was used to model the probability of illness for the three subpopulations including the healthy population, susceptible population and transplant recipients [51]. Finally, the number of people in a population of one million likely to get ill was generated from a binomial distribution. Sampling from the distributions were performed using $10^{6}$ simulations in the *R* package. This risk assessment model also enabled a prove of concept that a risk assessment assuming that *L. monocytogenes* grows as a single population characterized by an average relative $\mu_{max}$ value may either over or underestimate the risk of illness. To assess this, four cases were evaluated against the baseline situation where 50% of the strains belonged to both the susceptible and tolerant groups. The cases consisted of the case of only susceptible *L. monocytogenes* (Case 1), 25% susceptible versus 75% tolerant and 75% tolerant versus 25% susceptible. For each of these cases, the Spearman rank correlation was computed between the estimated number of cases per million consumers and increased in *L. monocytogenes* due to growth during consumer storage.

### 2.8. Data Availability

*L. monocytogenes* strain data including accession numbers for sequencing data and the stress phenotype data can be accessed online from Hingston et al. [24] at: https://www.ncbi.nlm.nih.gov/pmc/articles/PMC5340757/bin/Table1.XLSX. The data used in example risk assessment are available from the European Nucleotide Archive (ENA) under project number PRJEB15592.

## 3. Results

### 3.1. Bioinformatic Analysis

The number of core genes was 2258, while there were 5085 accessory genes making a total of 7343 genes in the pangenome. The matrix of percent similarity between the 7343 genes in the pangenome and the assembled *L. monocytogenes* genomes was generated for further use as input for machine learning predictive models.

### 3.2. Finite Mixture Modeling

Results on NPMLE diagnosis and the diagnosis for appropriateness of the number of components and adjustments where necessary are presented in Supplementary file (Section S1).

#### 3.2.1. Cold Stress Response

**Figure 4:**
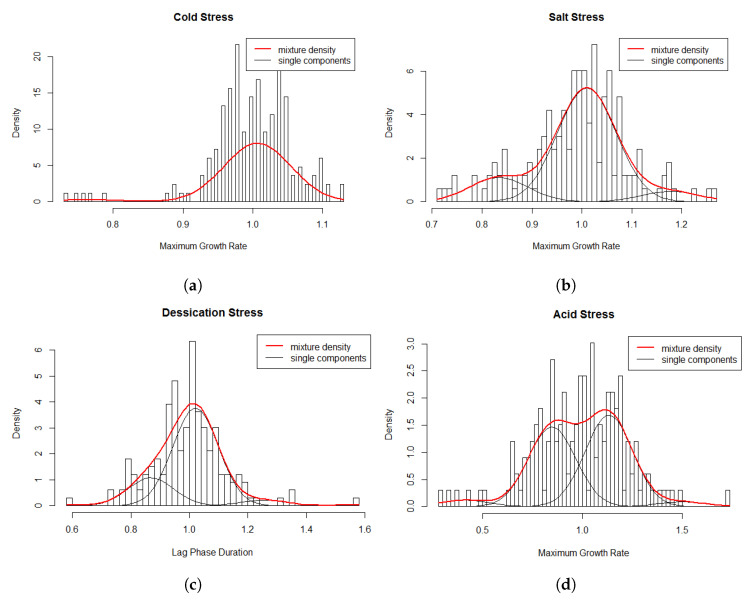
Histogram of relative growth rate parameters and the superimposed mixture model for the data.

Multi-modality in the relative $\mu_{max}$ values for the cold stress response was observed (Figure 3) suggesting that using the assumption of a homogeneous population would not be appropriate. A homogeneous normal distribution assumes an average relative $\mu_{max}$ 1.0 ± 0.06. The final model consisted of the following two-component model (Figure 4a): μ∼$\left(\begin{array}{c} \;0.76\;\;\;1.01\\ \;0.03\;\;\;0.97 \end{array}\right)$ which consists of a weighed sum of normal distributions:

Y∼0.03N(0.76,0.0023)+0.97N(1.01,0.0023).

A possible interpretation of the two components of the relative $\mu_{max}$ values of *L. monocytogenes* strains is shown in Table 2. Component one represents strains with the lowest average relative $\mu_{max}$ of 0.76 which consists of a small proportion (3%) of the strains. This population could be considered cold stress susceptible. Strains with the highest relative $\mu_{max}$ of 1.01 in component 2, consisting of a large majority of strains (97%) can be considered as cold stress tolerant.

#### 3.2.2. Salt Stress Response

The multi-modality in the relative $\mu_{max}$ values for salt stress response (Figure 3) indicated that using the assumption of a homogeneous population would not be appropriate. A homogeneous normal distribution assumes an average relative $\mu_{max}$ of 1.0 ± 0.1. The fitted model consisted of the following three-component (Figure 4b) approximation: μ∼$\left(\begin{array}{c} \;0.83\;\;\;1.01\;\;\;1.18\;\\ \;0.16\;\;\;0.77\;\;\;0.07\; \end{array}\right)$ which consists of a weighed sum of normal distributions with:

Y∼0.16N(0.83,0.0034)+0.77N(1.01,0.0034)+0.07N(1.18,0.0034).

Table 2 shows a possible interpretation of the three components of the relative $\mu_{max}$ values for response of *L. monocytogenes* strains to salt stress. The population in component one could be regarded as salt stress susceptible, component two as tolerant and component three as highly tolerant strains.

#### 3.2.3. Desiccation Stress Response

Multi-modality in relative LPD values for desiccation stress response (Figure 3) indicated that using the assumption of a homogeneous population may not be appropriate. A homogeneous normal distribution assumes an average relative LPD of 1.0 ± 0.13. The selected three-component model (Figure 4c) was: μ∼$\left(\begin{array}{c} \;0.87\;\;\;1.02\;\;\;1.26\;\\ \;0.21\;\;\;0.74\;\;\;0.04\; \end{array}\right).$ This three-component model consists of a weighed sum of normal distributions with:

Y∼0.21N(0.87,0.006)+0.74N(1.02,0.006)+0.04N(1.26,0.006).

A possible interpretation of the three components of the relative LPD values of *L. monocytogenes* strains response to desiccation stress is shown in Table 2. Component one consists of a population which could be termed as desiccation stress susceptible, component two as tolerant and component three as highly tolerant strains.

#### 3.2.4. Acid Stress Response

Multi-modality in the relative $\mu_{max}$ values for acid stress response indicated heterogeneity in relative $\mu_{max}$ for the *L. monocytogenes* strains (Figure 3). A homogeneous normal distribution assumes an average relative $\mu_{max}$ of 0.99 ± 0.23. The four-component model (Figure 4d) was: μ∼$\left(\begin{array}{c} \;0.41\;\;\;0.85\;\;\;1.13\;\;\;1.50\;\\ \;0.04\;\;\;0.44\;\;\;0.50\;\;\;0.03\; \end{array}\right).$ This four-component model consists of a weighed sum of normal distributions with:

Y∼0.04N(0.41,0.014)+0.44N(0.85,0.014)+0.50N(1.13,0.014)+0.03N(1.50,0.014).

The four components can be interpreted as shown in Table 2 by first considering the maximum and minimum relative $\mu_{max}$ values which are also biologically extremely high and low respectively (0.41 and 1.50). This is evident when these maximum and minimum component means for acid stress response were compared with the relative $\mu_{max}$ means of the highest and lowest components for growth under cold (0.76 and 1.01), salt (0.83 and 1.18) and desiccation (0.87 and 1.26) stress factors. Components one, two, three and four could be termed as highly susceptible, susceptible, tolerant and highly acid stress tolerant strains, respectively.

### 3.3. Predictive Modeling

#### 3.3.1. Data Pre-Processing

A total of 4959 of the 7343 genes in the pangenome were near zero variance predictors and the final predictor set consisted of 2384 genes. Initial modeling in the presence of class imbalances resulted in dismal class specific model performance which was remedied after up-sampling.

#### 3.3.2. Model Selection

The performance of the machine learning methods random forest (RF), support vector machine (SVM) (radial (SVMR) and linear kernels (SVML)), neural network (NN), stochastic gradient boosting (GBM) and logit boost (LB) was evaluated using the accuracy estimates from the 10-fold cross-validation. The class specific model performances were also evaluated (Supplementary File, Section S2). Table 3 presents the means, multiple comparison results and confidence intervals of the mean accuracy values. Kruskal–Wallis rank sum tests were used. Pairwise Mann–Whitney U-tests were conducted whenever the overall test indicated significant differences in model performances to evaluate which models significantly differed from the others while controlling the familywise error rate using the BH method [52].

For the acid stress response, SVMR had significantly higher mean accuracy (0.89; 95% CI: 0.88, 0.89) and κ of 0.91 and was selected for prediction of acid stress response category from the WGS predictors. RF with accuracy of (0.97; 95% CI: 0.95, 0.98) and κ of 0.98 was chosen for the prediction of cold stress response component from the WGS predictors. GBM and RF had significantly higher mean accuracies of 0.89 (95% CI: 0.87, 0.90) (Table 3) and a κ statistic of 0.98 and RF was selected for prediction of the salt stress response components from the WGS predictors. RF had significantly highest mean accuracy (0.91; 95% CI: 0.88, 0.92) (Table 3) and κ of 0.92 and it was therefore selected for prediction of the desiccation stress response category using WGS predictors.

### 3.4. Example *L. monocytogenes* Quantitative Risk Assessment

#### 3.4.1. Prediction of *L. monocytogenes* Stress Response Components

Supplementary Table S1 shows the predicted number of *L. monocytogenes* for each stress response type and component or sub-group within each of the response type for *L. monocytogenes* strains from different food types with unknown stress response components. The strains were classified into two of the four components of the acid response. A larger proportion of the strains 57% (n = 115) were acid stress tolerant while the rest of the strains were acid stress susceptible. Almost all (99%) of the strains were cold stress tolerant. A strikingly similar pattern was observed for salt and desiccation tolerance with 95% of strains being in the tolerant class. Two of the strains from meat and ready-to-eat foods were highly salt tolerant. The results from the dairy strains were used as input for the example microbial risk assessment on cultured dairy products.

#### 3.4.2. Quantitative Microbial Risk Assessment

Table 4 shows the estimated probability of illness, the number of estimated illnesses per million consumers from consumption of cultured milk products in three population groups and the increase in microbial counts during storage for the baseline situation as well as for the three scenarios. Estimated number of illnesses per million increased from zero (range: 0–3) to 2 (range: 0–33) and 790 (range: 4–23,653) for healthy population, susceptible population and transplant recipients, respectively. This increase corresponded with increased probability of illness per serving resulting from ordered increase in susceptibility from healthy, susceptible to transplant populations Table 4.

A homogeneous normal distribution assuming an average relative $\mu_{max}$ 1.0 ± 0.22 resulted in a higher estimated number of illnesses per million of zero (range: 0–4), 2 (range: 0–47) and 803 (range: 5–23,798) for healthy population, susceptible population and transplant recipients respectively. It should however be noted that the proportion of consumers in these subpopulations would also affect the national or population level risk estimates. For, instance, the healthy, susceptible and transplant populations were recently suggested to account for 76.7 %, 23.3 % and 0.0062%, respectively of the total population [53].

#### 3.4.3. Scenario Analysis

To perform scenario analysis, rank correlation assisted in establishing the degree to which large values of the estimated number of cases per million were associated with large values of increase in concentration of the pathogen during consumer storage. Spearman rank correlation was computed between the estimated number of cases per million and increase in concentration of the pathogen during consumer storage for the baseline scenario which involved 50% tolerant strains and changing the proportion of tolerant strains to 0%, 25% and 75%. The association was positive and increased with increase in the proportion of tolerant *L. monocytogenes* from 0%, 25%, 50% (baseline) with the highest proportion of tolerant strains of 75% showing highest positive association.

This trend was mirrored closely by the increase in concentration of the pathogen during storage for case 1, case 2 and case 3 which were 236 ± 139, 255 ± 150, 293 ± 172 cfu/g, respectively compared to 274 ± 161 cfu/g for the 50% tolerant proportion in the baseline situation.

## 4. Discussion

Microbial growth, a key input in exposure assessment during MRA, enables to estimate the concentration of ingested pathogenic microorganisms which is a key input for the calculation of the probability of illness from dose–response models. However, during exposure assessment, it is assumed that the species is a homogeneous unit despite evidence of a diverse microbial population structure and the associated increase in number of outbreaks. The first part of this study derives evidence that within each taxonomic unit population, there are sub-populations of varying proportions and with varying ability to grow under different stress conditions.

Refrigeration has been instrumental in decelerating the physical, microbiological and chemical spoilage of foods during all the stages of the food chain including processing, distribution, retail and domestic storage [54]. However, *L. monocytogenes* is recognized for its unique ability to survive and continue growing during refrigerated storage which increases risk of illness attributable to foods such as refrigerated, ready-to-eat (RTE) foods [24]. For the cold stress response, assuming a homogeneous normal distribution leads to an average relative $\mu_{max}$ of 1 ± 0.06. A two-component model was selected for the cold stress response. Almost all strains (97%) were cold tolerant with a high mean relative $\mu_{max}$ of 1 ± 0.002 for the tolerant group which was close to the one component arithmetic average relative $\mu_{max}$ of 1 ± 0.06. These findings are in agreement to the well-recognized ability of *L. monocytogenes* to grow at temperatures as low as minus 0.4 C [55].

A three-component approximation was appropriate for the salt stress response. A small proportion of the strains (7%) were in the component representing high tolerance relative $\mu_{max}$ (1.18 ± 0.0034) to salt stress. This increases concern because apart from enhancing palatability of food, salt remains part of the multiple hurdles of stress factors aimed at shelf life extension and improvement of microbial food safety by inhibiting or reducing growth of spoilage and pathogenic microorganisms [56]. Desiccation tolerance enables microorganisms to survive in food or on food contact surfaces for lengthy periods of time with little access to nutrients and water [57]. The mechanism of action of desiccation of foods on microorganisms is similar to that of salt stress due to a similar mode of action on microorganisms. Both stress factors function though reducing the amount of unbound water available for microbial growth in foods. One of the ways to achieve desiccation is the ability of sodium and chloride ions to associate with water molecules which induces a desiccation effect [56]. Similar to salt stress response, a three-component model for desiccation stress response was appropriate for this data.

The presence of organic acids or reduced pH either naturally inherent in foods or as process additives is a common inhibitor of microbial growth either alone or together with other process operations such as control of water availability, heating and cooling. Four components could be distinguished for acid stress response. It was unique for this stress response to find two almost equally prevalent subpopulations that were either highly susceptible (4%) or highly tolerant (3%) to acid stress. Their relative $\mu_{max}$ values were either biologically exceptionally low (highly susceptible subpopulation) or high (highly tolerant subpopulation) which was also evident from comparing these components with the mean relative $\mu_{max}$ for highest and lowest components of growth under cold, salt and desiccation stress phenotypes. Such high and low tolerance or susceptible strains for this and other stresses are of great interest in not only refining QMRA, but also for food process operators, control agencies and researchers investigating the biological mechanism underlying the persistence of certain *L. monocytogenes* strains in the food processing environment [24]. Such strains have posed challenges due to their persistence despite efforts to inactivate them either through processing of foods or cleaning and disinfection of food process operations.

Changes in genetic composition of microorganisms often encode for large phenotypic differences in growth, survival and inactivation [9]. Furthermore, genes are transferred between and within bacterial species thus adding heterogeneity in growth within the taxonomic unit. WGS data could therefore be highly discriminatory predictors or even act as biomarkers discriminating between different levels of resistance to stress conditions. However, many applications of WGS data for predicting phenotypes have been hindered by challenges in the translation of high dimensional WGS data into reduced phenotypic information with a resultant metric that is useful in MRA [13]. The second aim of this study was to select predictive algorithms inputting highly dimensional WGS data for predicting into which of the *g* sub-populations new strains with unknown stress response data lies by treating the *g* sub-populations as classes. If the sub-population is predicted for new strains, the proportion of each sub-population can then be calculated while the mean and variance of the relative $\mu_{max}$ or relative LPD for each of these populations is computed from the finite mixture models.

SVMR was selected for prediction of acid stress response while RF was chosen for cold, salt and desiccation stress responses. RF, a tree-based approach, has a number of attractive properties. Decision trees commonly suffer from high variance where for instance two randomly split training datasets would result in quite different results if a decision tree is fit to each halve [35]. Bootstrap aggregation or bagging reduces this variance in the context of decision trees by obtaining many training sets from the population. Separate prediction models are built from each of the training sets and eventually the resulting predictions are averaged. These ML algorithms are increasingly applied in biology research areas such as gene expression, tissue classification, gene function prediction, protein subcellular location prediction, protein secondary structure prediction and protein folding prediction [58].

The final part of this study constructed an example consumer level QMRA of cultured milk products with strains of unknown stress response phenotypes to illustrate the phenotype class prediction and role of *L. monocytogenes* strain stress phenotype heterogeneity in contributing to differing risk of illness. The first part of the QMRA involved predicting for each strain the stress response sub-group where it lies for each of the stress factors and different food categories using the selected ML models. As commonly known for *L. monocytogenes*, almost all (99%) of the strains were predicted to be in the cold stress tolerant component. A similar pattern was observed for salt and desiccation tolerance with 95% of strains being in the tolerant class. These two stress factors share similar mechanisms of microbial inhibition and the similarities in predictions are therefore in agreement to well-grounded principles of food preservation [56]. Acid stress response phenotype is important in cultured milk products where organoleptic properties, safety and shelf-stability are dependent on lowering of the pH to around 4.6 which increases the acidity [22]. The strains from cultured milk products used in this QMRA were classified into a mixture of two normally distributed components each at 50% of the total population leading to an equivalent number of both acid tolerant and susceptible strains.

The probability of illness per serving as well as expected number of cases per million consumers increased with increased susceptibility from healthy population, susceptible population to transplant recipients. This is in agreement with increased probability of a single bacterial cell to cause listeriosis in more susceptible consumer groups [51]. The estimated number of illnesses per million increased from zero (range: 0–3) to 2 (range: 0–33) and 790 (range: 4–23,653) for healthy population, susceptible population and transplant recipients, respectively. This increase corresponded with increased probability of illness per serving resulting from increased susceptibility for the three consumer populations. These estimates are within the ranges of those estimated in report on risk characterization of Listeria monocytogenes in ready-to-eat foods by the Joint FAO/WHO Expert Consultation on Risk Assessment of Microbiological Hazards in Foods who estimated the number of cases ranging from 0.01 to 1580 at consumption exposures ranging of −1.5–7.5 (Log CFU/serving) [51]. It should however be considered that the estimates from the FAO/WHO report concerned ready-to-eat foods in general and the cultured milk in our case study is just a subset of ready-to-eat foods.

Assuming a homogeneous normal distribution where the relative $\mu_{max}$ is the arithmetic average from the data resulted in a higher estimated number of illnesses per million in the three consumer sub-populations in comparison to the model taking into account that the strains consisted of a mixture of two normally distributed components each containing 50% of both tolerant and susceptible strains with different mean relative $\mu_{max}$ values. We therefore demonstrate that use of population average growth parameters resulted in overestimation of risk estimates from the QMRA. There were sharp contrasts in number of strains classified under each stress response phenotype between our findings and those of Hingston et al. [24] in their analysis which assumed a homogeneous normal distributed population of strains. For instance, we found that 51 strains were tolerant to all the stress phenotypes while the study of Hingston et al. [24] found none, 64 strains were tolerant to 3 stress types while Hingston et al. [24] found 2. Fifteen strains were tolerant to two stress phenotypes while Hingston et al. [24] found 24 which is a higher number of strains. None of the strains were susceptible to all the 3 stress phenotypes while Hingston et al. [24] found 5. This reflects differences in proportions of tolerant and susceptible strains as well as between relative $\mu_{max}$ values of single [24] and mixture distributions (this study). This illustrated that assuming that the strains growing consist of a homogeneous normal distributed population of strains may overlook the underlying phenotypic stress subpopulations.

To assess the sensitivity of the risk to the proportion of strains in each component, Spearman rank correlation was computed between the estimated number of cases per million and increase in concentration of the pathogen during consumer storage for the baseline scenario of 50% tolerant and change in proportion of tolerant strains to 0%, 25% and 75%. We found out that increase in the proportion of tolerant *L. monocytogenes* resulted in increased association between the estimated number of cases per million and increase in concentration of the pathogen during consumer storage. This can be attributed to the increase in concentration of the pathogen during storage for the scenarios involving 0%, 25% and 75% tolerant proportion groups which were 236±139,255±150, 293±172 cfu/g, respectively compared to 274±161 for the 50% tolerant proportion.

The approach demonstrated in this study is of potential practical benefit to research, food industry and regulators in addressing the bottleneck to the application of WGS for exposure assessment during QMRA. An important benefit will be the reduction in uncertainty in EA models and the corresponding reduction in uncertainties when making risk estimates. A major milestone towards more accurate decision making at improved reaction times will be the ability to conduct exposure assessment using WGS in microbial strains where growth phenotypic data are not available. Use of WGS data in predictive modeling to draw conclusions beyond data obtained will foster models supporting reduced need for frequent use of slow culture dependent laboratory tests and food validation of growth, survival and inactivation models under differing conditions. The use of WGS for exposure assessment also supports the need for timely detection of shifts in bacterial stress tolerance arising from genetic changes. This will improve public protection and mitigation through a dynamic MRA process which maintains higher resolution despite changes in microbial genetic composition. The modeling protocol will support a more straightforward construction of user friendly online platforms whose performance improve with time in a similar way to many areas where machine learning methods have found application. However, the practical application will involve the collection of a database of strains with available WGS and phenotypic data on microbial adaptation to various inherent food characteristics and conditions encountered during food processing and handling. This will enhance the generalizability of the predictions by accounting for genetic variation in microbial populations. The results from this study enhance the potential to conduct complete farm to fork MRA using WGS data when used in combination with recently reported approaches where machine learning and WGS were used for predicting risk of illness [33] and to improve hazard characterization in microbial risk assessment [34].

## 5. Conclusions

**Figure 5:**
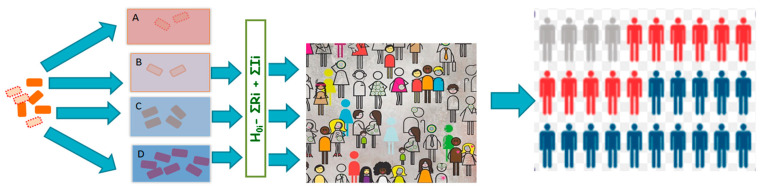
Overview of conclusions: Heterogeneous pathogen events. Pathogen populations (A–D) with stochastically varying phenotypes enter various biotic and abiotic environments. Extreme environments kill pathogens in environment (A), while favorable conditions support vigorous pathogen growth in the environment (D). Intermediate environments support pathogen survival (B) or moderate growth (C). Various surviving pathogen subsets emerging from the various events result in risk variation in an often heterogeneous host population whose susceptibilities vary leading to stochasticity in disease risk.

Results from our study demonstrate that reliance on growth parameters derived from population average assumes that the pathogen acts as a uniform taxonomic unit which neglects within-species heterogeneity in microbial stress response. Figure 5 presents an illustrative summary of the proposals and findings from this study. Neglecting within-species heterogeneity in microbial stress response may compromise the resolution of QMRA and the quality of evidence used for subsequent infection control efforts. This is because microbial growth, which is a key input quantity in exposure assessment, enables the estimation of the concentration of ingested pathogenic microorganisms which is a key input for the calculation of probability of illness from dose-response models. Heterogeneity in the growth rates has an impact on variation in survival of the pathogen in the environment, food and ultimately through the human host barriers. Neglecting this heterogeneity hinders the predictive accuracy of risk assessment efforts.

The rapid throughput of WGS sequencing data compared to laboratory growth studies will support a future where predictive models based on WGS data greatly reduce the need for future validation of models in the laboratory and in food.

## Figures and Tables

**Table 1:** Summary of the listeriosis quantitative risk assessment model for consumption of cultured milk at domestic level: variables, equations or distribution of the input parameters and data sources.

Variable/Parameter	Description	Value/Equation	Distribution	Unit	Data Source
$C_{o}$	Initial concentration	$10^{3}$ to $10^{4}$	Uniform	cfu/g	[22]
$T_{sl}$	Storage time	Minimum 0.5, and	Pert	Days	[22]
		Most likely: 6 to 10			
		Maximum: 45			
$\mu_{maxSusce}$ ( $\mu_{maxTol}$)	Maximum growth rate for susceptible (tolerant) *L. monocytogenes*	Mean: 0.762 ± 0.047 (1.007 ± 0.047)	Normal	per hour	Calculated
Serving	Portion consumed	Mean: 236.75 ± 170	Log normal	gram	[22]
Hold	Increase during storage	$\mu_{maxSusce}*T_{sl}+\left(\mu_{maxTol}\right)*T_{sl}$		cfu/g	This study and [49] model for exponential growth phase
D	Ingested dose	Serving × ($C_{o}$+ Hold)		cfu/serving	Calculated
$r_{h}$	Dose–response parameter for healthy subpopulation	2.37 × $10^{-14}$		−	[51]
$r_{s}$	Dose–response parameter for susceptible subpopulation	1.06 × $10^{-12}$		−	[51]
$r_{t}$	Dose–response parameter for healthy subpopulation	5.8 × $10^{-10}$		−	[51]
$P_{illH}$	Probability of illness for healthy subpopulation	1−exp${}^{\left(-r_{h}*D\right)}$	Exponential	−	Exponential dose–response model [51]
$P_{illS}$	Probability of illness for susceptible subpopulation	1−exp${}^{\left(-r_{s}*D\right)}$	Exponential	−	Exponential dose–response model [51]
$P_{illT}$	Probability of illness for transplant subpopulation	1−exp${}^{\left(-r_{t}*D\right)}$	Exponential	−	Exponential dose–response model [51]
$N_{illH}$	Number illness per million servings for healthy subpopulation	$B(1000000,P_{illH})$	Binomial	−	Calculated
$N_{illS}$	Number illness per million servings in susceptible subpopulation	$B(1000000,P_{illS}$	Binomial	−	Calculated
$N_{illT}$	Number illness per million servings for transplant subpopulation	$B(1000000,P_{illT})$	Binomial	−	Calculated

**Table 2:** Probabilities, averages of categories and interpretations of the L.monocytogenes stress response categories.

**Cold**				**Acid**			
**Component**	$\pi_{j}$	$\mu_{j}$	**Interpretation**	**Component**	$\pi_{j}$	$\mu_{j}$	**Interpretation**
1	0.03	0.76	Susceptible	1	0.04	0.41	Highly Susceptible
2	0.97	1.01	Tolerant	2	0.44	0.85	Susceptible
				3	0.50	1.13	Tolerant
				4	0.03	1.50	Highly Tolerant
**Salt**				**Desiccation**			
**Component**	$\pi_{j}$	$\mu_{j}$	**Interpretation**	**Component**	$\pi_{j}$	$\mu_{j}$	**Interpretation**
1	0.16	0.83	Susceptible	1	0.21	0.87	Susceptible
2	0.77	1.01	Tolerant	2	0.74	1.02	Tolerant
3	0.07	1.18	Highly Tolerant	3	0.04	1.26	Highly Tolerant

**Table 3:** Machine learning model performance for prediction of *L. monocytogenes* stress response categories.

	Stress Type *							
Model	Acid		Cold		Salt		Desiccation	
GBM	0.87 ${}^{abc}$	(0.83–0.89)	0.97 ${}^{a}$	(0.96–0.98)	0.89 ${}^{a}$	(0.87–0.90)	0.89 ${}^{ab}$	(0.86–0.90)
RF	0.87 ${}^{ab}$	(0.86–0.88)	0.97 ${}^{a}$	(0.95–0.98)	0.89 ${}^{a}$	(0.87–0.90)	0.91 ${}^{a}$	(0.88–0.92)
SVMR	0.89 ${}^{c}$	(0.88–0.89)	0.97 ${}^{a}$	(0.96–0.98)	0.83 ${}^{b}$	(0.81–0.84)	0.83 ${}^{c}$	(0.80–0.84)
SVML	0.85 ${}^{a}$	(0.84–0.87)	0.96 ${}^{a}$	(0.94–0.97)	0.85 ${}^{b}$	(0.83–0.86)	0.88 ${}^{ab}$	(0.86–0.90)
NN	0.72 ${}^{d}$	(0.68–0.78)	0.96 ${}^{a}$	(0.93–0.98)	0.63 ${}^{c}$	(0.57–0.68)	0.69 ${}^{d}$	(0.56–0.76)
LB	0.89 ${}^{bc}$	(0.88–0.90)	0.97 ${}^{a}$	(0.97–0.98)	0.85 ${}^{ab}$	(0.83–0.88)	0.86 ${}^{bc}$	(0.85–0.88)

* Mean (range); means within a column with similar lower case superscript letter are not significantly different; random forest (RF), support vector machine (radial (SVMR) and linear (SVML) kernels), gradient boosting (GBM), neural network (NN) and logit boost (LB) models.

**Table 4:** Results for quantitative microbial risk assessment of *L. monocytogenes* at the consumer level in cultured milk products.

	Minimum	1st Quartile	Median	Mean	3rd Quartile	Maximum
Number ill healthy per million	0	0	0	0	0	3
Number ill susceptible per million	0	0	1	2	2	33
Number ill transplant per million	4	321	585	790	1019	23653
Probability of illness healthy $\times (10^{-6})$	0.00	0.01	0.02	0.03	0.04	0.98
Probability of illness susceptible $\times (10^{-4})$	0.00	0.01	0.01	0.01	0.02	0.44
Probability of illness transplant $\times (10^{-1})$	0.00	0.00	0.01	0.01	0.01	0.24
Increase during storage: all susceptible (cfu/g)	9	127	215	236	326	803
Increase during storage: 75 % susceptible (cfu/g)	10	137	232	255	352	867
Increase during storage: 50 % susceptible (cfu/g)	11	147	250	274	378	933
Increase during storage: 25 % susceptible (cfu/g)	11	157	267	293	404	994

## Supplementary Materials

The following are available online at https://www.mdpi.com/2076-2607/8/11/1772/s1.
