# Supplementary material for: Quantitative Microbial Risk Assessment Based on Whole Genome Sequencing Data: Case of Listeria monocytogenes
Source: Microorganisms. 2020 Nov 11;8(11):1772. doi: 10.3390/microorganisms8111772 (PMC7698238; doi:10.3390/microorganisms8111772)
Supplement: Supplementary file 1 [file microorganisms-08-01772-s001.pdf]

Supplementary file

Quantitative microbial risk assessment based on whole genome sequencing data: case of *Listeria monocytogenes*

Patrick Murigu Kamau Njage[1,*], Pimlapas Leekitcharoenphon[1], Lisbeth Truelstrup Hansen[2], Rene S. Hendriksen[1], Marc Aerts[3], Christel Faes[3], Tine Hald[1]

[1]Technical University of Denmark, National Food Institute, Research Group of Genomic Epidemiology

[2]Technical University of Denmark, National Food Institute, Research Group for Microbiology and Hygiene

[3]Interuniversity Institute for Biostatistics and statistical Bioinformatics, Hasselt University Katholieke Universiteit Leuven, Belgium

[*]panj@food.dtu.dk

Contents

1 Finite mixture modeling **3**

2 Predictive Modeling **5**

3 Example *L. monocytogenes* quantitative risk assessment **6**

4 R code **7**

1 Finite mixture modeling

Designation of stress response phenotype components

To diagnose if the solution for $\hat{G}$ is NPMLE, the gradient functions $d(G, p)$ for the mixing distribution G were plotted. The first condition stipulates that $\hat{G}$ is NPMLE when for all support points x, the gradient functions $d(\hat{G}, x)$ are less than one. Secondly, if $\hat{G}$ is NPMLE, $d(\hat{G}, x)$ reaches one at all support points x of $\hat{G}$. The last condition is that $\hat{G}$ has support points within the interval $[a, b]$ such that all the x functions $f_i(y_i|x)$ have unique modes observable in the plot of the gradient function (Schlattmann, 2009).

Cold stress response

Checking for parsimony, a homogeneous one-component solution was computed and compare with the two-component model. The parametric bootstrap approach yielded a p-value of $<$ 0.001 indicating that a two-component model seems to be appropriate for these data on the basis of the use of the LRS. Testing for a three-component model against the two-component model yielded a simulated p-value of 0.27 indicating a negligible decrease in the log likelihood. The two-component model was selected. Figure 1 shows grid point G versus the gradient function $d(G, P)$ to diagnose the NPMLE. G was NPMLE because $d(G, P) \leq 1$ in the μ_{max} interval $[0.73, 1.13]$. $d(G, P)$ was also 1 for p in $0.76, 1.01$. Finally, the estimate from the model was unique because the gradient function was not identically one.

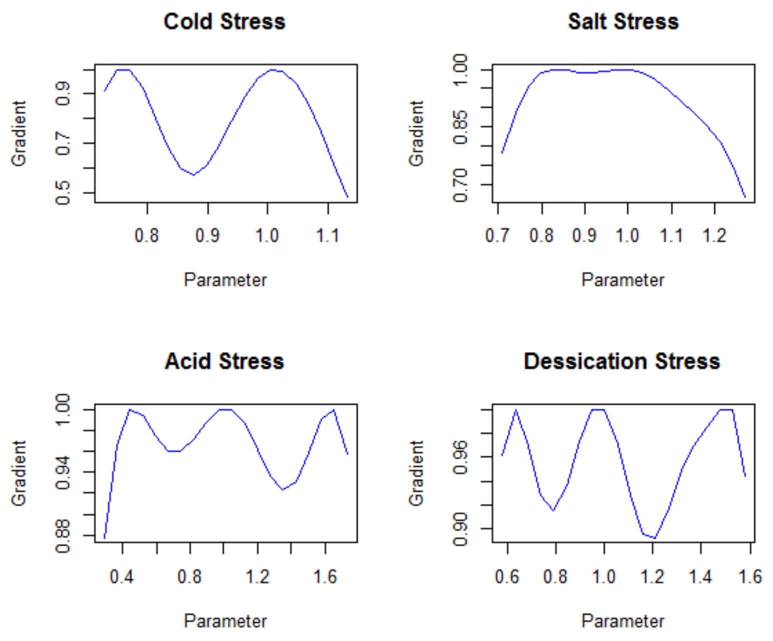

Figure (1) Grid point G versus gradient function $d(G, P)$ for microbial growth data under cold, salt, acid and desiccation stress

Salt stress response

The fitted model suggested a three-component approximation with Log-Likelihood at maximum of 152.56 (Figure ??). To assess parsimony, a two-component solution was computed and compared with the three-component model. The parametric bootstrap approach yielded a p-value of 0.012 indicating for these data a three-component model seems to be appropriate on the basis of the use of the LRS. Figure 1 (Appendix 1) shows grid point G versus the gradient function $d(G, P)$ to diagnose the NPMLE. G was NPMLE because $d(G, P) \leq 1$ in the μ_{max} interval $[0.71, 1.3]$. G also reaches approximately one at all support points and has support points between the minimum and maximum maximum growth rate.

Desiccation stress response

The fitted model suggested a five-component approximation with Log-Likelihood at maximum of 118.5. There were extremely small weights for the first (0.6%) and last components (0.6%) which was an initial motivation that a three-component mixture may be considered. Further assessment was performed by backward selection where four, three and two component solutions were fitted and evaluated. The parametric bootstrap LRS test yielded p-values > 0.05 comparing five against four component mixtures and four against three. However comparing a three-component model against the two-component model yielded a simulated p-value < 0.001 indicating for these data a three-component model seems to be appropriate (Figure ??). Figure 1 (Appendix 1) shows grid point G versus the gradient function $d(G, P)$ to diagnose the NPMLE. G was NPMLE because $d(G, P) \leq 1$ in the μ_{max} interval $[0.58, 1.58]$. G also reaches approximately one at all support points and has support points between the minimum and maximum maximum lag phase duration.

Acid stress response

The fitted model suggested a four-component approximation with Log-Likelihood at maximum of 15.58. Comparing a three-component model against the four-component model yielded a simulated p-value < 0.05 indicating for these data a four-component model seems to be appropriate. Figure 1 (Appendix 1) shows grid point G versus the gradient function $d(G, P)$ to diagnose the NPMLE. G was NPMLE because $d(G, P) \leq 1$ in the μ_{max} within the data range interval. G reaches approximately one at all support points and has support points between the minimum and maximum maximum growth rate.

2 Predictive Modeling

Model Selection

The performance of the machine learning methods random forest (RF), support vector machine (SVM) (radial (SVMR) and linear kernels (SVML)), neural network (NN), stochastic gradient boosting (GBM) and logit boost (LB) was evaluated using the accuracy estimates from the 10-fold cross-validation. In order to choose the statistical hypothesis test approach, assumptions of analysis of variance (ANOVA) namely, that the data are normally distributed and there is homogeneity of variance across the the model accuracy were assessed (Kurtner, Nachtsheim, 2009). Diagnostic results and those of class specific model performances are presented here.

Acid Stress

The plot of residuals versus fitted values indicated no relationships between residuals and fitted values therefore implying homogeneity of variance. Levene's test however showed evidence that the variance across groups was statistically significantly different ($p < 0.01; F = 4.13, 5df$). Plotting the quantiles of the residuals against the quantiles of the normal distribution showed heavy tails away from the reference line indicating that the assumption of normal distribution was not met. This conclusion was supported by the Shapiro-Wilk test on the ANOVA residuals ($W = 0.82, p < 0.001$).

Kruskal-Wallis rank sum test was used followed by pairwise Mann–Whitney U-tests while controlling the familywise error rate using the BH method by Benjamini and Hochberg (1995). Sensitivity (specificity) values for prediction of highly susceptible, susceptible, tolerant and highly tolerant class for the selected model, SVMR, were 1(1), 0.92(0.98), 0.93(0.97) and 1(1) respectively. Positive predictive values (negative predictive values) from prediction highly susceptible, susceptible, tolerant and highly tolerant classes by SVMR model were 0.99(1), 0.95(0.97), 0.92(0.98) and 0.99(1) respectively.

Cold Stress

Assessing the homogeneity of variance assumption using a plot of residuals versus fitted values indicated no evident relationships between residuals and fitted values. Levene's test indicated that the variance across groups was not statistically significantly different ($p > 0.05; F = 1.27, 5df$). Plotting the quantiles of the residuals against the quantiles of the normal distribution showed heavy tails, an indication that the assumption of normal distribution was not met. Shapiro-Wilk test on the ANOVA residuals was significant ($W = 0.94, p < 0.01$) confirming that the assumption of normal distribution was not met.

Sensitivity, specificity, positive predictive and negative predictive values for the selected RF model were 1, 0.98, 0.98 and 1 respectively.

Salt Stress

The plot of residuals against fitted values depicted no relationships between residuals and fitted values which implied homogeneity of variance. Levene's test however showed evidence that the variance across groups was statistically significantly different ($p < 0.001; F = 7.1, 5df$). When quantiles of the residuals were plotted against the quantiles of the normal distribution, heavy tails were observed indicating possible violation of the ANOVA assumption of normal distribution. This conclusion was supported by Shapiro-Wilk test on the ANOVA residuals ($W = 0.95, p < 0.05$). Kruskal-Wallis rank sum test was used followed by pairwise Mann–Whitney U-tests while controlling the familywise error rate using the BH method by Benjamini and Hochberg (1995).

Sensitivity (specificity) values from prediction of susceptible, tolerant and highly tolerant classes by using RF were 1(0.98), 0.96(1) and 1(1) respectively. Positive predictive values (negative predictive values) from prediction susceptible, tolerant and highly tolerant classes by using RF model were 0.96(1), 1(0.98) and 1(1) respectively.

Desiccation Stress

The plot of residuals versus fitted values depicted no relationships between residuals and fitted values implying homogeneity of variance. Levene's test however showed evidence for heterogeneity of variance in accuracy values across the models ($p < 0.05; F = 2.7, 5df$). The Q-Q plot showed heavy tails giving evidence that the assumption of normal distribution was not met which was also confirmed by a significant Shapiro-Wilk test on the ANOVA residuals ($W = 0.75, p < 0.001$). Kruskal-Wallis rank sum test was used followed by pairwise Mann–Whitney U-tests while controlling the familywise error rate using the BH method by Benjamini and Hochberg (1995).

Sensitivity, specificity, positive and predictive values of the class predictions for susceptible, tolerant and highly tolerant from the RF model were all 1.

3 Example *L. monocytogenes* quantitative risk assessment

Prediction of *L. monocytogenes* stress response components

Table 1 shows the predicted number of *L. monocytogenes* for each stress response type and component or sub-group within response type for *L. monocytogenes* isolates from different food types with unknown stress response components.

Table (1) Number of isolates in each stress response phenotype class predicted using machine learning models for new food isolates with unknown stress phenotypes

	Acid				Cold		Salt			Desiccation		
Food Category	HS	S	T	HT	S	T	S	T	HT	S	T	HT
Dairy	0	18	19	0	0	37	1	36	0	3	34	0
Meat	0	20	24	0	0	44	1	42	1	2	42	0
Fish	0	16	19	0	1	34	2	33	0	1	34	0
Ready to eat	0	10	21	0	0	31	1	29	1	2	29	0
Vegetables	0	13	13	0	1	25	2	24	0	1	25	0
Mix food	0	9	19	0	0	28	1	27	0	1	27	0

Highly susceptible (HS), susceptible (S), tolerant (T), highly tolerant (HT)

4 R code

```
#Codes for cold stress example are given
###########################################################
###Finite mixture modeling example of cold stress         #
###########################################################

#df: Cold.stress
>head(Cold.stress)
  colname Umax
1       1 0.73
2       2 0.75
3       3 0.76
#Histogram of cold stress maximum growth rates
x <- Cold.stress$Umax
par(mfrow=c(2,2))
hist(x, prob=TRUE, col="grey", xlab="Umax", main="Cold Stress", breaks=20)#
prob=TRUE for probabilities not counts
lines(density(x), col="blue", lwd=2) # add a density estimate with defaults
lines(density(x, adjust=2), lty="dotted", col="darkgreen", lwd=2)

#Fit finite mixture model combining both VEM and EM
library(CAMAN)
npml<-mixalg(obs="Umax", family="gaussian", data=Cold.stress, acc=10^(-8),
numiter=50000, startk=50)
```

```
##################
#Parametric bootstrap tests: two versus one component
em1<-mixalg.EM(npml,p=c(0.96933114),t=c(0.7622953))
em2<-mixalg.EM(npml,p=c(0.03066886, 0.96933114),t=c(0.7622953,1.0066507))
ll<-anova(em0,em2,nboot=250) #might take some minutes

#two versus 3
em3<-mixalg.EM(npml,p=c(0.03066886, 0.96933114,0.041970285),t=c(0.7622953,1.0066507,
1.2550669))

ll<-anova(em2,em3,nboot=250) #might take some minutes

#Classification of L. monocytogenes isolates into components of the mixture
emcold<-mixalg.EM(obs="Umax", family="gaussian",data=Cold.stress,
              t=c(0.7622953,1.0066507), p=c(0.03066886,0.96933114), acc=10^(-20))
#Plotting gradient function
m0 <- mixalg.VEM(npml, family="gaussian",data=Cold.stress,startk=20)
plot(m0@totalgrid[,2],m0@totalgrid[,3], type="l",xlab="Parameter",ylab="Gradient",
col = "blue", main="Cold Stress")

###################################################
###Machine learning example of cold stress        #
###################################################


library("caret")
#Specifies amount of cores R can use in order to make it run faster
library(doParallel)
#Find out how many cores are available
cores<-detectCores()
#Create cluster with desired number of cores, leave one free
#core processes
cl <- makeCluster(cores[1]-1)
#Register cluster
registerDoParallel(cl)
#
# Data preprocessing
#
#Near zero variance remove
nzv <- nearZeroVar(df_ML1)
filteredDescr <- df_ML1[, -nzv]
df_ML1 <- filteredDescr
```

```
#Attach outcome /cold phenotype classes from finite mixture models to the isolates:
#should be sorted in same order
Cold <- Cold.df$Cold
listacid <- cbind(Cold, df_ML1)
#
#Model selection: Class Imbalanced Dataset
#

# randomly pick 70% of the number of observations
index <- sample(1:nrow(df_ML1),size = 0.7*nrow(df_ML1))

# subset  to include only the elements in the index
training <- df_ML1[index,]

# subset  to include all but the elements in the index
testing <- df_ML1[-index,]

#Exploration of outcome variable "Cold".

qplot(Cold,data=training, main="Distribution of Cold phenotypes")
+theme(axis.text=element_text(size=14, color="black"))+
  theme(axis.title=element_text(face="bold",size="14"))
#The cross validation. number = 10
fitCtrl <- trainControl(method = "cv",number = 10, verboseIter = F)

# generate dataframe over multiple prediction
predDf <- data.frame(run = 0, time = 0, gbm = 0, rf = 0, svmr = 0,
svml = 0, nn=0, lb = 0)
#```
##Running the ML algorithms
#```{r}
start.time.all = Sys.time() #log the starting time
# Run the model buiding 10 times & record accuracy over test set
for (i in 1:10){
  index <- sample(1:nrow(df_ML1),size = 0.7*nrow(df_ML1))
# subset  to include only the elements in the index
training <- df_ML1[index,]
# subset  to include all but the elements in the index
testing <- df_ML1[-index,]
  dim(training)
  dim(testing)
  #Start building model
```

```
  start.time = Sys.time()
  mod.gbm <- train(Cold~ . , data= as.data.frame(training), method = "gbm",
  trControl = fitCtrl, verbose = F)
  mod.rf <- train(Cold~ . , data= training , method = "rf",
  trControl = fitCtrl, verbose = F)
  mod.svmr <- train(Cold~ . , data= training , method = "svmRadial",
  trControl = fitCtrl, scale = FALSE, verbose = F)
  mod.svml <- train(Cold~ . , data= training , method = "svmLinear",
  trControl = fitCtrl, scale = FALSE, verbose = F)
  mod.nn <- train(Cold~ . , data= training , method = "nnet",
  trControl = fitCtrl, MaxNWts=85000, verbose = F)
  mod.lb <- train(Cold~ . , data= training , method = "LogitBoost",
  trControl = fitCtrl, verbose = F)
  stop.time = Sys.time()

  #Predictions
  pred_val <- c( i, (stop.time - start.time),
unname(confusionMatrix(predict(mod.gbm, testing), testing$Cold)$overall[1]),
unname(confusionMatrix(predict(mod.rf, testing), testing$Cold)$overall[1]),
unname(confusionMatrix(predict(mod.svmr, testing), testing$Cold)$overall[1]),
unname(confusionMatrix(predict(mod.svml, testing), testing$Cold)$overall[1]),
unname(confusionMatrix(predict(mod.nn, testing), testing$Cold)$overall[1]),
unname(confusionMatrix(predict(mod.lb, testing), testing$Cold)$overall[1]))
  predDf <- rbind(predDf, pred_val)
}
stop.time.all = Sys.time()
#calculate total time for execution
print(stop.time.all - start.time.all)

#correct the prediction frame
predDf <- predDf[-1,]
##Accuracy of the models
#Following displays the accuracy of the six models for all runs.
#Models are referred by short names.

library(knitr)
rownames(predDf) <- NULL
kable(predDf[,-c(2)], digits = 3)
#Average accuracy of all runs for all models are as per following

modAccuracy <- data.frame(colMeans(predDf[,-c(1,2)]))
colnames(modAccuracy) <- "Avg. Accuracy"
```

```
kable(t(modAccuracy), digits = 3)
#
#Confidence intervals
#algorithm bcanon of bootstrap R package.
#df
> head(listcold)
  gbm    rf  svmr  svml    nn    lb
1 0.948 0.927 0.948 0.969 0.927 0.969
2 0.969 0.979 0.969 0.948 0.979 0.979
algorithm <- function(x){bcanon(x,1000,mean(x),alpha=c(0.025,0.975))}
#Confidence intervals
set.seed(123)
coldBCaCI <- apply(listcold, 2, algorithm)

#"Out of sample" accuracy
validAccuracy <- data.frame(Accuracy = c(
  confusionMatrix(predict(mod.rf, testing), testing$Cold)$overall[1],
  confusionMatrix(predict(mod.nn, testing), testing$Cold)$overall[1],
  confusionMatrix(predict(mod.gbm, testing), testing$Cold)$overall[1],
  confusionMatrix(predict(mod.lb, testing), testing$Cold)$overall[1],
  confusionMatrix(predict(mod.svml, testing), testing$Cold)$overall[1],
  confusionMatrix(predict(mod.svmr, testing), testing$Cold)$overall[1]))
rownames(validAccuracy) <- c("rf", "nn", "gbm", "lb", "svml", "svmr")
kable(t(validAccuracy), digits = 3)
#
# Unbalanced dataset high model accuracies but class
#specific accuracies are quiet dismal
#
#Upsampling
#
set.seed(123)
up_train <- upSample(x =df_ML1, y = df_ML1$Cold)
table(up_train$Class)
  S   T
160 160
#Rename
df_ML1 <- up_train
library("dplyr")
df_ML1 <- rename(df_ML1, Cold = Class)
#Rerun ML model selection process
#Confusion Matrix and Statistics
confusionMatrix(predict(mod.rf, testing), testing$Cold)
```

```
#
#Model selection: hypothesis testing
#
>head(listcold)
  model accuracy
1   gbm    0.948
2   gbm    0.969
3   gbm    0.958

# Compute the analysis of variance
res.aov <- aov(accuracy ~ model, data = listcold)
# Summary of the analysis
summary(res.aov)

#Check ANOVA assumptions
# 1. Homogeneity of variances
plot(res.aov, 1, main="Cold stress")
# Levene's test,
library(car)
leveneTest(accuracy ~ model, data = listcold)

# 2. Normality
plot(res.aov, 2, main="Normal probability plot cold stress",
xlab="Theoretical Quantiles")

#Shapiro-Wilk test
# Extract the residuals
aov_residuals <- residuals(object = res.aov )
# Run Shapiro-Wilk test
shapiro.test(x = aov_residuals )

#Non-parametric alternative to one-way ANOVA test
# Kruskal-Wallis rank sum test,
kruskal.test(accuracy ~ model, data = listcold)

#If above significant
#Multiple pairwise-comparison between groups with BH
corrections for multiple testing.

PTcold <- pairwise.wilcox.test(listcold$accuracy,
listcold$model, p.adjust.method = "BH")
```

```
####################################################
###Quantitative microbial risk assessment         #
####################################################
#
#Prediction of components for new isolates: example
#of isolates from dairy foods
#
listfinpreddairy <- listfinpred[ which(listfinpred$ ‘Food matrice‘==’Diary’), ]
listfinpreddairy <- as.data.frame(listfinpreddairy[,-c(1:3)])

listcoldpredictiondairy <- predict(finMod.rf, listfinpreddairy)
#####################
#QMRA Baseline
#############################
sims<-1000000
##Initial Listeria Concentration: range given:
uniform distribution
#
conc_o <- runif(sims, 1000, 10000)
#Storage time (days): min: 0.5, most likely: 6 to 10, maximum: 45. Below in hours
mini <-0.5*24
modest <- runif(sims, 6*24, 10*24)
maxest <- 45*24
tsl <- rpert(sims, min=mini, mode=modest, max=maxest, shape=4) #Time storage

#Increase during holding: Buchanan model for exponential growth phase
# hold = Umax*(storagetime) which is composed of 50%
proportion of two growth components
# Change proptol and propsuscept for scerario analysis so that
# Case 1: 0% propsuscept, Case 2: 25% propsuscept versus 75% proptol,
#Case 3: 75% proptolversus 25% propsuscept
propsuscept <- 0.5
proptol <- 0.5
umaxsuscept <- rnorm(sims, 0.7622953, 0.00228928537495403)*propsuscept
umaxtol <- rnorm(sims, 1.0066507, 0.00228928537495403)*proptol
hold <- umaxtol*tsl+umaxsuscept*tsl

#Portion consumed
#Mean 236.75 g, var: 170^2
m <- 236.75
```

```
var <- 170^2
par1 <-par1<-log(m/sqrt(1+var/m^2))
par2<-sqrt(log(1+var/m^2))
quantperserving <-rlnorm(sims,meanlog=par1,sdlog=par2)

#Final concentration
Cfinal <- conc_o+hold

#Dose per serving
D <- quantperserving*(conc_o+hold)

#Probability of illness per serving
#Dose response : exponential Pill(D;r)=1-exp^(-r*D) for 3 populations
#Where r = 2.37*10^-14 for healthy; 1.06*10^-12 fpr susceptible and 5.8*10^-10
#for transplant recipients

Pill_healthy=1-exp(-2.37*10^-14*D)
Pill_susceptible=1-exp(-1.06*10^-12*D)
Pill_transplant=1-exp(-5.8*10^-10*D)
#Expected number of cases per million servings
#Generate how many persons in the population of 1000 000 get
#infected from a binomial distribution

Cases_PerMil_healthy <- rbinom(sims,1000000, Pill_healthy)
Cases_PerMil_susceptible <- rbinom(sims,1000000, Pill_susceptible)
Cases_PerMil_transplant <- rbinom(sims,1000000, Pill_transplant)


# Rank correlation of inputs for different cases

#Healthy
rh<-c(cor(Cases_PerMil_healthyav,holdav,method="spearman"),
cor(Cases_PerMil_healthy,hold,method="spearman"),
 cor(Cases_PerMil_healthy1,hold,method="spearman"),
     cor(Cases_PerMil_healthy2,hold2,method="spearman"),
     cor(Cases_PerMil_healthy3,hold3,method="spearman"))
barplot(rh,horiz=TRUE,names.arg=c("Growth baseline","Growth case 1", "Growth case 2",
"Growth case 3"), col = "lightgreen", main="Healthy population")
#Repeat for susceptible and tolerant
```
